# Supplementary material for: Nanoarchitectonics of Fe-Doped Ni3S2 Arrays on Ni Foam from MOF Precursors for Promoted Oxygen Evolution Reaction Activity
Source: Nanomaterials (Basel). 2024 Sep 4;14(17):1445. doi: 10.3390/nano14171445 (PMC11397559; doi:10.3390/nano14171445)
Supplement: Supplementary file 1 [file nanomaterials-14-01445-s001.zip › nanomaterials-3153845-supplementary.pdf]

Supporting Information

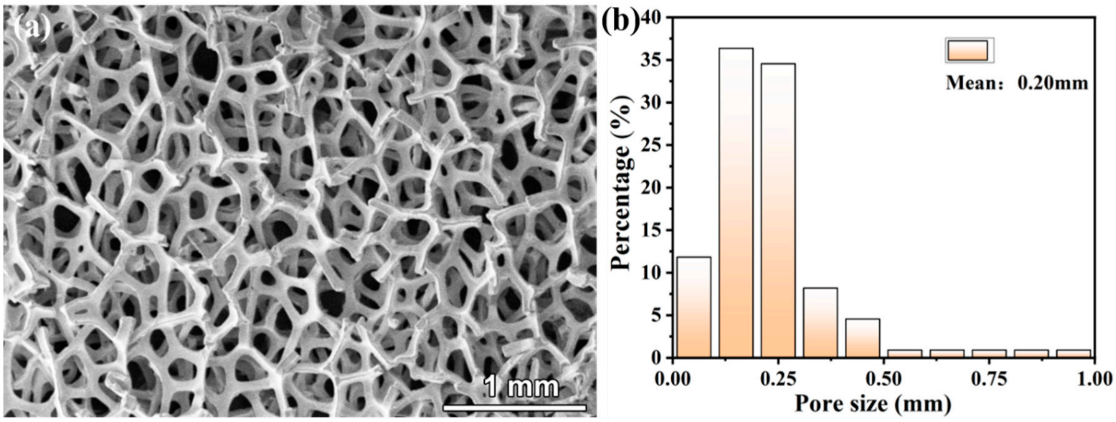

Figure S1. (a) The SEM image of Ni Foam, (b) the corresponding pore size distribution.

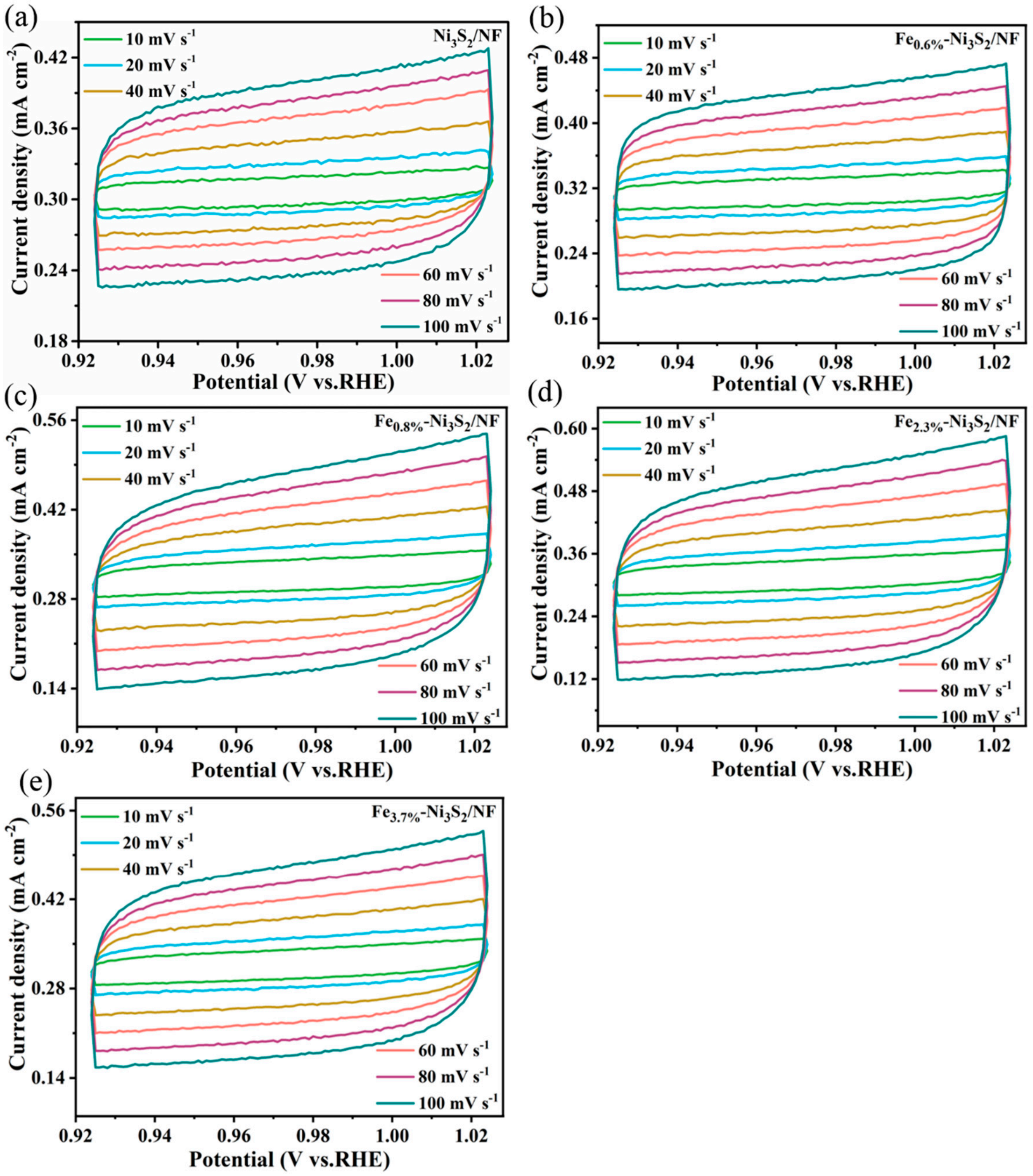

Figure S2. CV curves of (a) Ni_3S_2/NF, (b) $Fe_{0.6\%}$-Ni_3S_2/NF, (c) $Fe_{0.8\%}$-Ni_3S_2/NF, (d) $Fe_{2.3\%}$-Ni_3S_2/NF and (e) $Fe_{3.7\%}$-Ni_3S_2/NF at different scan rates (10 ~ 100 mV s^{-1}).

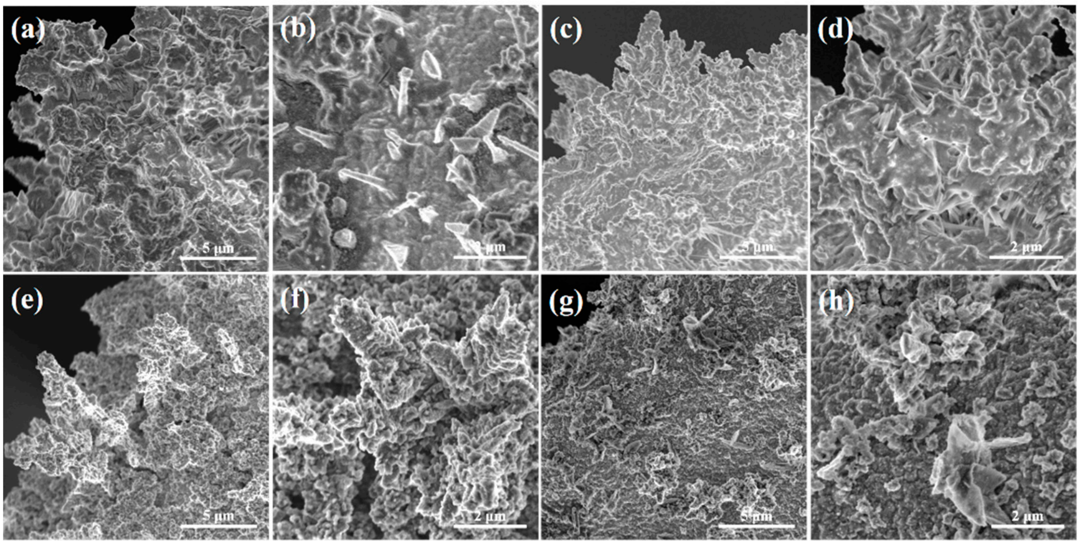

Figure S3. SEM images of (a-b) $Fe_{0.6\%}$-Ni_3S_2/NF, (c-d) $Fe_{0.8\%}$-Ni_3S_2/NF, (e-f) $Fe_{2.3\%}$-Ni_3S_2/NF, (g-h) $Fe_{3.7\%}$-Ni_3S_2/NF after stability tests.

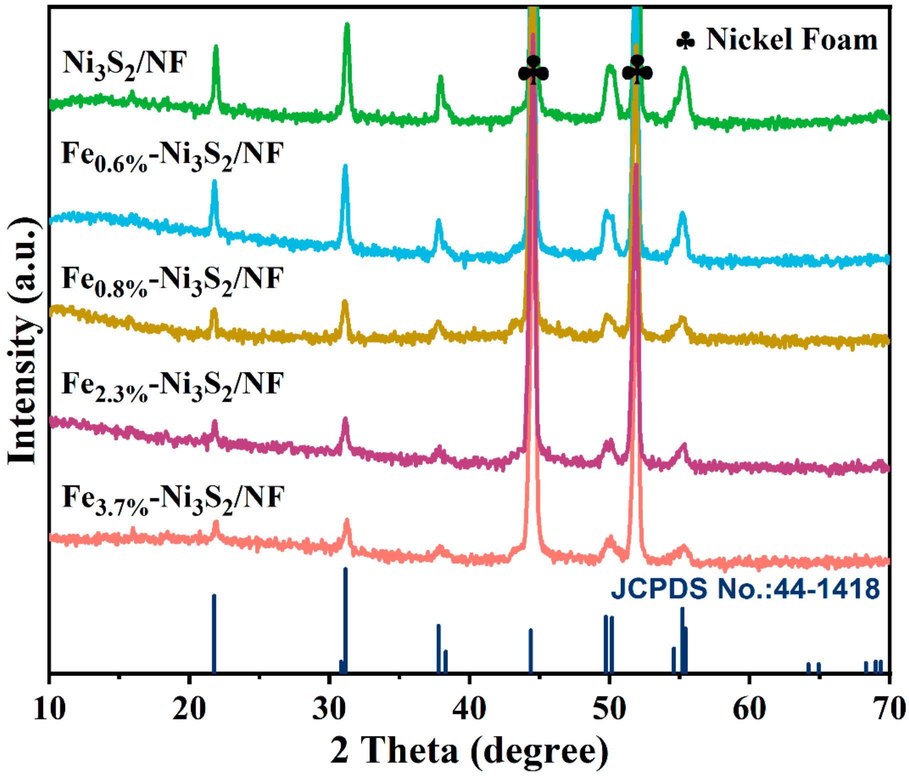

Figure S4. PXRD patterns of Ni_3S_2/NF, $Fe_{0.6\%}$-Ni_3S_2/NF, $Fe_{0.8\%}$-Ni_3S_2/NF, $Fe_{2.3\%}$-Ni_3S_2/NF and $Fe_{3.7\%}$-Ni_3S_2/NF electrodes after OER stability tests.

Table S1 The comparison of XPS peak values before and after the stability tests.

$Fe_{2.3\%}$-Ni_3S_2/NF Catalysts	Ni 2p 3/2	Ni 2p 1/2	Fe 2p3/2	Fe 2p3/2	S 2p 3/2	S 2p 1/2
Ni^{2+}	852.52	856.06				
Ni^{2+}(12 h)	852.90	856.61				
Ni^{3+}	856.06	873.78				
Ni^{3+}(12 h)	856.61	874.35				
Fe^{2+}			706.05			
Fe^{2+} (12 h)			706.50			
Fe^{3+}				712.53		
Fe^{3+} (12 h)				713.31		
S^{2-}					162.15	163.49
S^{2-} (12 h)					162.50	163.79
